# Effect of ethanol osmotic dehydration on CO_2_ puffing and drying mechanism of potato

**DOI:** 10.1016/j.fochx.2023.100715

**Published:** 2023-05-18

**Authors:** Yao Niu, Haifeng Chen, Zifeng Zhang, Yuejin Yuan, Shaobo Dong, Zhuo Xu

**Affiliations:** Shaanxi University of Science and Technology, Xi'an, China

**Keywords:** Osmotic dehydration, Puffing drying, Ethanol (PubChem CID:702), Pretreatment, CO_2_ (PubChem CID:280)

## Abstract

In order to further improve the drying quality of potato slices, this study investigated the effects of EPD (CO_2_), HAD + EPD (CO_2_), EH + EPD (CO_2_), and FD on the quality and physicochemical properties of potato slices. The changes in solid loss (SL), obtained ethanol (OE), water loss (WL), and moisture content under different ethanol concentrations and soaking times were studied. The effects of WL, SL, OE, and moisture on puffing characteristics were also studied. The results show that in the EH + EPD (CO_2_) process, the use of ethanol and CO_2_ as the puffing media improves the puffing power. WL and OE have significant effects on hardness, crispness, expansion ratio, and ascorbic acid. The quality of potato slices puffed and dried by ethanol osmotic dehydration is better, which provides a new method for potato slice processing.

## Highlights

• EH + EPD (CO_2_) has good structures, colors, and high ascorbic acid contents, and possesses moderate crispness and hardness.
• Based on the Peleg model, EH + EPD (CO_2_) has a great ability to absorb water and retain water.
• It explained the phenomenon of mass transfer of potatoes in different concentrations of alcohol at different times.
• The effects of WL, OE, SL, moisture, puffing pressure difference and puffing temperature on the drying quality and physicochemical properties of CO_2_ high-pressure low-temperature puffing-dried potato chips were studied.

## Graphical abstract

**Figure:**
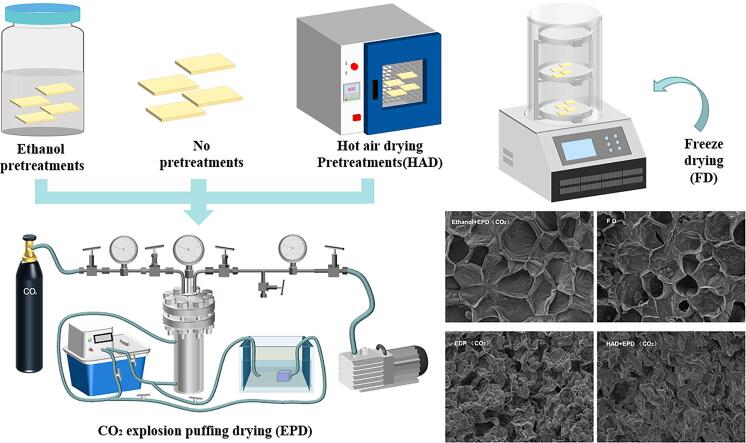

## Introduction

Solanum tuberosum is the fourth largest crop in the world, after rice, wheat, and maize in the total production (Zhu et al., 2021). Potato tubers contain starch, rich in protein and amino acids, which can provide rich calories for the human body, and there are a variety of vitamins (vitamin C, vitamin A, vitamin B1, B2 and vitamin B6) and minerals, especially the types of vitamins that are the most complete of all food crops (Wu et al., 2018). Fresh potatoes have a short dormancy period, short shelf-life, and high moisture content, so they are prone to spoilage. Drying is an important method to prolong the shelf-life of potatoes for processing, transportation, storage, and use (Wang et al.,2020).

Drying is a physical unit operation that involves both heat and mass transfer, the last of which is usually the rate-limiting factor (Rojas et al., 2019). At present, there are various drying methods, and the different drying methods affect the quality and flavor of the product. Hot air drying is an ancient and widely used process to reduce the moisture content of foods and improve the shelf-life and stability, but prolonging exposure to high temperatures can result in poor product quality and higher-energy consumption (Mokhtar et al., 2019). Freeze-drying maximizes the retention of quality of the original material and bioactive components, but it is a time-consuming and energy-intensive process, and the dried product is a non-brittle, spongy structure (Zhu et al., 2022). Contrary to the drying methods described above, expansion puffing drying (EPD) is a way of creating a porous, crispy product structure (Lyu et al., 2017). Its principle is to put fresh fruits and vegetables through different pretreatments, following the phase change principle and the gas thermocompression effect, to put the material in the puffing chamber, and then to change the temperature and pressure so that the moisture inside the processed material is instantly gasified and evaporated, and relying on the expansion of the gas is to drive the denaturation of the material structure in the tissue so that the material forms a uniform porous structure, which has a particular expansion ratio and crispness (Lyu et al., 2021). It has been reported that combined with CO_2_ puffing drying, the samples obtained by hot air drying pretreatment have better overall acceptance, textural properties, low shrinkage and high crispness (Song et al., 2020). Many kinds of pretreatments can be taken before puffing and drying. At present, there is hot air drying, vacuum drying, freeze drying, etc., but few papers report that ethanol treatment is used as a pretreatment method for CO_2_ puffing and drying. Ethanol is less harmful to the human body and has virtually no residue after drying, which can considerably improve the drying process and the quality of the food product (Granella et al., 2022).

Before this study, few scholars have studied the method of puffing and drying after ethanol pretreatment, and have not explored its principle. To improve the quality of dried potato products, this study evaluated CO_2_ expansion puffing drying (EPD (CO_2_)), hot air drying pretreatment followed by CO_2_ expansion puffing drying (HAD + EPD (CO_2_)), ethanol pretreatment followed by CO_2_ expansion puffing drying (EH + EPD (CO_2_)) and freeze-drying (FD) potato slices in terms of color, rehydration ratio, expansion ratio, microstructure, texture characteristics, and ascorbic acid content. The puffing and physicochemical properties of potato slices were studied from the effects of different ethanol concentrations and soaking time on WL, SL, OE and moisture content. The effects of WL, OE, SL, and moisture content on the puffing processes were investigated.

## Material and methods

### Raw materials

Fresh potatoes were purchased at a local market (Xi’an, Shaanxi, China). The average initial moisture content was 574.69 ± 30% (dry basis) or 80 ± 2% (wet basis). Potatoes of good quality with no deterioration were selected. And the process included washing, peeling, and slicing into 45 × 45 × 2 mm^3^ pieces by a sharp slicer (Homemade Food Slicer, Xi’an, Shaanxi, China).

### Pretreatment

#### Ethanol pretreatments (EH)

Potato slices were immersed in ethanol solutions of different concentrations for a period of time at 25 °C, and the solid–liquid ratio was 1:8 (g/ml). In a comparison of several different drying methods, the ethanol pretreatment concentration is 30% and the soaking time is 20 min. During the ethanol osmotic dehydration process, ethanol was used to replace the water in the potato slices. The weight of the potato changed, including water loss (WL), solid loss (SL), and obtained ethanol (OE). The soaking method was divided into single-stage soaking (the ethanol concentrations were 15%, 30%, 50%, and 75%, respectively) and multi-stage soaking (the ethanol concentrations were 15%+30%, 30%+50%, 15%+30%+50%, 15%+50%, 15%+30%+50%+75%, 30%+50%+75%, 50%+75%, 15%+30%+75% and each stage of the soaking time was equal) in different ethanol concentration soaking, the different total soaking time (10, 15, 20, 25, 30, 35, 40, 45, and 50 min) to explore the mass transfer phenomenon of potato samples during ethanol pretreatment. A food-grade paper towel was used to absorb the remaining ethanol solution on the surface of the slices and then weighed to calculate the weight on a wet basis.

#### Hot air drying pretreatments (HAD)

The potato slices were put into an electric constant temperature blower box (DHG-9070A, Shanghai Yiheng Scientific Instrument Co., Ltd., Shanghai, China) for pretreatment. The hot air drying experiments carried out at a constant air velocity (2 m/s) and at an air temperature of 60 ℃. The moisture of treated slices were weighed down to 30% on a wet basis. All pretreatment experiments were repeated three times.

### Explosion puffing drying process (EPD)

**Fig. 1:**
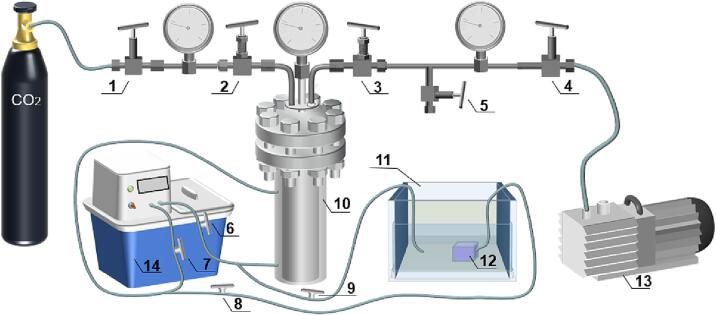
CO_2_ explosion puffing dryer and related units: 1. safety valve; 2. inlet needle valve; 3. outlet needle valve; 4. connection valve; 5. pressure relief valve; 6. hot water inlet valve; 7. hot water outlet 8. cooling water outlet valve 1; 9. cooling water inlet valve; 10. puffing chamber; 11.cooling water tank; 12. circulating water pump; 13. vacuum pump; 14. constant temperature water tank.

The puffing experiments were conducted using a self-made high-pressure low-temperature air flow puffing and drying equipment system (Fig. 1), which consisted of a puffing chamber, a vacuum pump, a carbon dioxide cylinder, a constant temperature water tank, a cooling water tank, and pressure gauges. During the puffing process, hot water was injected at a constant temperature to reach a predetermined temperature, and then the pretreated material was put in the puffing chamber and the cover was tightened. A constant pressure of CO_2_ was filled into the puffing chamber, and it was maintained for 15 min to balance the internal and external pressures of the sample, and the carbon dioxide gas was fully penetrated into the material. Then, the valve was opened for just a moment before it got closed again, so as to release the pressure in the puffing chamber to 0.1 MPa. After that, the vacuum pump was connected to the puffing chamber. The pressure of the puffing chamber was rapidly changed from the state of high pressure to the state of vacuum and vacuum drying. After ten minutes, the material was cooled in the puffing chamber and formed a sample. Next, we measured the sample characteristic parameters of hardness, crispness, expansion ratio, and ascorbic acid, and then the samples were sealed in the polyethylene bags.

### Freeze-drying process (FD)

Freeze-drying was conducted using a vacuum freeze dryer (TF-LFD-1, Shanghai Tianfeng Industrial Co., Ltd., Shanghai, China). Fresh potato slices were first pre-frozen in a refrigerator (DW-86L, Haier, Qingdao, China) at −72 ± 2 °C for 6 h, and then quickly placed in the freezer dryer. The temperatures of vacuum freeze-drying were divided into eight sections of −40 °C, −30 °C, −20 °C, −10 °C, 0 °C, 10 °C, 20 °C, and 30 °C, respectively for two hours each. And the operating pressure of vacuum freeze dryer was 1 Pa.

### Analytical methods

#### Determination of mass transfer

The quality changes of potato slices included WL, SL, and OE. The water loss ratio (WL), solid loss ratio (SL), and ethanol obtained ratio (OE) of slices at different ethanol concentrations and soaking methods were calculated. Moisture content was determined gravimetrically by drying the samples in a vacuum at 60 °C until a constant weight was reached (Wang et al., 2019). The experiments of each group with the same variables were repeated for 5 times to get these data. The experiment used a single factor approach, and before studying one factor, the other variables were fixed. In mass transfer calculations, the alcohol meter (CJM-3526, Hebei, China) was used to measure the proportion of alcohol in the solution after soaking. The conversion relationship between density and volume of ethanol was obtained according to the comparison table of relative density and content of ethanol, and the water loss (WL), solid loss (SL) and obtained ethanol (OE) of potatoes within a certain period of time (10, 15, 20, 25, 30, 35, 40, 45, and 50 min) were obtained by the following equation.

(2.1) $$\rho =\frac{\rho_{c}V-m_{G}}{V}$$

(2.2) $$\mathrm{WL}\left(\%\right)=\frac{M_{1}-M_{w}}{m_{1}}=\frac{M_{2}-M_{e}-M_{w}}{m_{1}}\times 100\%$$

(2.3) $$\mathrm{SL}\left(\%\right)=\frac{m_{G}}{m_{g1}}=\frac{m_{g1}-m_{g2}}{m_{g1}}\times 100\%$$

(2.4) $$\mathrm{OE}\left(\%\right)=\frac{m_{e}}{m_{1}}=\frac{M_{0}-M_{e}}{m_{1}}\times 100\%$$

where *ρ* was the corrected density of the solution after soaking, *ρ*_c_ was the measured density of the solution after soaking, *V* was the volume of the solution after soaking, *m*_G_ was the solid loss mass of the samples after soaking, *M*_1_ was the moisture mass of the solution after soaking, and *M*_w_ was the initial moisture mass of the solution before soaking, *M*_2_ was the total mass of the solution after soaking, *M*_0_ was the ethanol mass of the solution before soaking, *M*_e_ was the ethanol mass of the solution after soaking, *m*_e_ was the mass of potato slices after soaking, *m*_1_ was the initial mass of the potato slices, *m*_g1_ was the dry matter mass before pretreatment, *m*_g2_ was the dry matter mass after pretreatment.

The moisture content after ethanol treatment (*M_P_*), including the water remaining and obtained ethanol, was calculated with the following equation.

(2.5) $$M_{P}\left(\%\right)=\frac{m_{2}-m_{g2}}{m_{g2}}\times 100\%$$

where *m*_2_ was the mass of potato slices after pretreatment, and *m*_g2_ was the dry matter mass after pretreatment.

#### Determination of color

The color of the potato slice samples was measured using a spectrophotometer (an Intelligent Spectrophotometer, Shenzhen Sansishi Technology Co., Ltd., Shenzhen, China). The color properties of fresh (reference) and dried potato samples were measured in terms of CIELAB parameters. Before each experiment, a whiteboard and a blackboard were used to calibrate. The sample measurement was repeated five times, and the sample measuring points were randomly selected (Li et al., 2019).

(2.6) $$\Delta E=\sqrt{{\left({\text{a}}_{0}^{*}-{\text{a}}_{1}^{*}\right)}^{2}+{\left({\text{b}}_{0}^{*}-{\text{b}}_{1}^{*}\right)}^{2}+{\left(L_{0}^{*}-L_{1}^{*}\right)}^{2}}$$

where L* was the brightness, a* was the red/green, and b* was the yellow/blue. Δ*E* was the total color difference between fresh and dried potato slices.

#### Determination of texture

Flat potato slices were chosen and measured with a physical property analyzer (TA. XTPLUS, Stable Micro Systems, UK). The test conditions were as follows: TPA double-cycle compression mode; the speed before the test was 1.0 mm/s; the test speed was 0.5 mm/s; the speed after the test was 5.0 mm/s; the test distance was 2.0 mm/s; the probe was P/2N; and the trigger force was 5.0 g. The maximum force represented the hardness, and the crispness was the first peak force of the broken sample (Zhang et al., 2017). The data obtained by measuring five groups of samples were averaged and recorded.

#### Determination of expansion ratios

Expansion ratios (ER) were evaluated in the different sample volumes before and after expansion. Based on the principle of the volume replacement method, the volume of potato slices was measured by filling it with ultra-fine quartz sand, which was calculated according to the following equation.

(2.7) $$\mathrm{ER}=\frac{V_{1}/m_{1}-V_{0}/m_{0}}{V_{0}/m_{0}}$$

where *V*_1_ was the volume after puffing (cm^3^), *V*_0_ was the volume before puffing (cm^3^), *m*_1_ was the mass after puffing (g), and *m*_0_ was the mass before puffing (g).

#### Determination of rehydration ratios

The rehydration ratio (RR) was a method to study the degree of cellular damage during drying. Each dried slice was immersed in 40 mL of 25 ± 1 °C distilled water for rehydration. The change in the sample moisture over time was then determined by mass balance. The slices were measured after taking out of the water periodically. The excess water on the surface was wiped off with absorbent paper, weighed, and put back into the water until the mass was a constant (Wang et al., 2018).

(2.8) $$\mathrm{RR}=M_{R}/M_{D}$$

where *RR* was the rehydration ratio; *M_R_* was the mass after rehydration (g); *M_D_* was the mass before rehydration (g).

#### Determination of ascorbic acid

The ascorbic acid content in each 100 g of potatoes could be determined using the titration method, and the reagent was 2,6-dichloroindophenol (Song et al., 2020). When the titrated potato solution was changed from colorless to reddish, all ascorbic acids was oxidized, and the ascorbic acid content (VC) could be calculated according to the following formula (Yen et al., 2008).

(2.9) $$\mathrm{VC}=\frac{V\times (V_{1}-V_{0})\times \rho }{V_{V}\times m}\times 100\%$$

where VC was the ascorbic acid per 100 g of potato slices (mg/100 g); *V_V_* was the volume of the titration solution (cm^3^); *m* was the total mass of the samples (g); *V* was the total volume of the sample solution (cm^3^); *V*_1_ was the volume of dye consumed when the potato solution (cm^3^) was titrated; *V_0_* was the volume of dye consumed when titrating for the control group (cm^3^); *ρ* was the mass of ascorbic acid corresponding to 1 mL of the dye solution (mg).

#### Scanning electron microscopy (SEM)

A scanning electron microscopy was used to analyze the microstructural characteristics of the samples by using a VEGA 3 SBH SEM at 300 × magnification and 10 kV voltage. The sample was mounted on the SEM stub to provide a reflective surface of the electron beam, and gold was sprayed in a vacuum using a sputter coater. The representative photomicrographs were selected for each sample to study (Yuan et al., 2022).

### Mathematical description

The rehydration data were fit by using the Peleg model (Eq. 10) (Peleg, 1979), where *M*(t) was the moisture content on a dry basis at time *t* (min) (*g* water/g dry matter), *M_0_* was the initial moisture content, *k*_1_ was the rate constant (min·d.b^-1^), and *k*_2_ was the asymptotic level constant (d.b^-1^). The reciprocal of *k*_1_ represented the water absorption rate, and the reciprocal of *k*_2_ represented the water retention capacity.

(2.10) $$M\left(t\right)=M_{0}+\frac{t}{k_{1}+k_{2}t}$$

The parameters of each model were interactively adjusted to the experimental data by minimizing the sum of squared errors between the experimental and predicted values (SSE in Eq. 11), which implemented in the function fitting in MATLAB R2019b.

(2.11) $$\mathrm{SSE}=\sum_{i=1}^{x}{\left(\left(predicted\right)-\left(experiemental\right)\right)}_{i}^{2}$$

### Statistical analyses

Statistical analyses of data were done by using IBM SPSS 26.0 software (IBM SPSS, USA), and each data was the mean (±SD) of multiple replicates. Comparison between groups was made using one-way ANOVA based on the Tukey test, and differences in means were demonstrated to be significant at p < 0.05. The data were analyzed by Origin 2021b software and MATLAB R2019b software, and the test results were drawn.

## Results and discussion

### Effects of different pretreatments of potato slices

#### Physicochemical qualities

Color is a significant sensory parameter of food after drying, and it is also one of the important indicators to attract consumers to buy. Higher values of L*, a* and b* represent the increased lightness, redness, and yellowness. The L* values of FD dried potato slices are increased compared with those of fresh samples, the a* and b* values of potato slices after EPD, EH + EPD, and HAD + EPD are significantly increased (P < 0.05), among which the a* value of HAD + EPD is the highest, and the b* value of EPD is the highest. The L* of the EH + EPD is the highest, and the Δ*E* value of the EH + EPD is the lowest (Table 1). The increased a* value may be due to discoloration from enzymatic and non-enzymatic browning reactions, loss of ascorbic acid and pigment during HAD pretreatment. A study by Gracia-Martinez *et al.* pointed out that the decrease in L* value and the increase in a* value both reflect the browning of the product during drying (García-Martínez et al., 2013). Compared with other pretreatment methods, the L* of EH + EPD is higher. According to the research of Feng, it may be due to the decolorization effect of ethanol, which leads to the dissolution of pigments, and the whitening of the tissues of ethanol-pretreated samples (Feng et al., 2019). According to Wang *et al.*, ethanol can inhibit the enzymatic browning reaction (Wang et al., 2019). Carbon dioxide can also prevent the material from oxidizing and cause browning in EPD, so the Δ*E* value of EH + EPD is the lowest.

**Table 1:** Effect of different drying methods on physicochemical qualities of potato.

Quality indicator	Fresh	FD	EPD(CO_2_)	EH + EPD(CO_2_)	HAD + EPD(CO_2_)
L*	84.89 ± 0.05^b^	99.87 ± 0.02^a^	70.84 ± 0.29^c^	94.97 ± 0.13^ab^	68.99 ± 0.92^c^
a*	−2.32 ± 0.03^d^	−2.53 ± 0.12^d^	0.33 ± 0.06^b^	−1.63 ± 0.05^c^	0.69 ± 0.03^a^
b*	11.83 ± 0.04^c^	13.55 ± 0.04^bc^	28.52 ± 0.06^a^	16.17 ± 0.07^b^	24.78 ± 0.06^a^
*ΔE*		15.08 ± 0.09^b^	21.98 ± 0.03^a^	10.99 ± 0.07^c^	20.73 ± 0.70^a^
Hardness(g)		1931.23 ± 135.59^c^	2123.12 ± 245.61^c^	5410.14 ± 236.85^a^	4823.23 ± 256.61^b^
Crispness(g)		1032.21 ± 244.68^c^	1123.13 ± 253.17^c^	2697.49 ± 349.07^a^	1836.53 ± 399.33^b^
ER		0.99 ± 0.04^c^	1.16 ± 0.03^b^	1.55 ± 0.04^a^	1.09 ± 0.05^c^
Ascorbic acid	24.91 ± 0.48^a^	20.46 ± 1.08^b^	15.06 ± 0.77^d^	18.39 ± 1.23^c^	11.28 ± 0.89^e^

*Note*: Different letters in the same row indicate significant differences among pretreatment (p < 0.05).

The crispness of the product is also the main factor for consumers to pursue food. The crispness is generally determined by the degree of softness and hardness. The hardness, crispness, and expansion ratio can well reflect the taste of potato slices. The hardness and crispness of FD dried potato slices are lower than those of EPD, EH + EPD and HAD + EPD. FD-dried foods have high-quality characteristics, such as high porosity and low shrinkage. During the FD process, the frozen water in the material is directly sublimated from the solid phase to the gas phase, so that the porous structure can be well maintained inside (Chen et al., 2017). The dried EH + EPD (CO_2_) slices have the largest crispness and hardness, followed by HAD + EPD (CO_2_), and EPD (CO_2_) dried slices are the smallest (Table 1). During the EPD (CO_2_), carbon dioxide is dissolved in the moisture of the raw material under high pressure. The material is suddenly released from the high-pressure state in a vacuum, and the carbon dioxide carries the moisture to evaporate, which enhances the puffing power, resulting in a product with high crispness and high expansion ratio. In HAD + EPD (CO_2_), the structure of the material dried by HAD + EPD is denser (Xu et al., 2022). The surface of the pretreated material is dense and the hardness is high, and the proportion of carbon dioxide infiltration is small, resulting in relatively low expansion power, a higher bulk density and greater hardness. The solubility of carbon dioxide in ethanol is greater than that in water, and more carbon dioxide can be dissolved in the expansion medium, which increases the expansion power, the expansion ratio, the crispness and the hardness.

Ascorbic acid is a nutrient that is vital to human growth and development (Rueda Kunz 2021). The ascorbic acid contents of potato slices are as follows: Fresh > FD > EH + EPD > EPD > HAD + EPD (Table 1). There are a lot of factors that affect the loss of ascorbic acid, including pH, temperature, oxygen, light, and enzyme activity (Santos * Silva, 2008). Exposure of the samples to air during HAD pre-treatment results in more loss of ascorbic acid. It can be intuitively observed that the ascorbic acid content of EH + EPD is higher than that of EPD in Table 1. Wang *et al.* found that the ethanol absorbed by green onions could minimize the contact between water and ascorbic acid, and avoid dissolution and oxidation in water. EPD potato slices without pretreatment have a high moisture content, which results in oxidation and dissolution of some ascorbic acid in water. Carbon dioxide is used in the pressurization process of the EH + EPD. Carbon dioxide is an acid oxide, and ascorbic acid is relatively stable in an acidic environment, so that the ascorbic acid content with FD is p < 0.05.

#### Microstructure

**Fig. 2:**
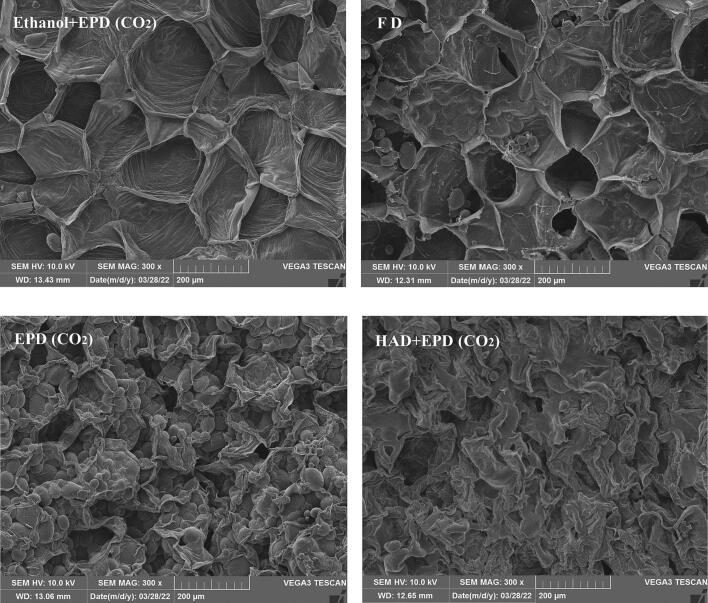
SEM of samples with different drying methods.

The SEM images of potato slices obtained by FD, EPD, EH + EPD, and HAD + EPD are shown in Fig. 2. The SEM image obtained by EPD shows that there are many complete cells and honeycomb structures in the potato slices. The pressure is quickly released when the material is overheated. Relied on the puffing power of carbon dioxide during the puffing process, the structures of the polymer and other substances inside the potato slices are denatured, so the slices form a honeycomb structure. The pre-drying method affects the pore number and size, which can disrupt capillary channels and volume expansion (Feng et al., 2021). The SEM images of HAD + EPD show that the potato slices have severe cell shrinkage and compact structure, resulting in irregular pores. A homogeneous honeycomb structure is observed in the SEM images of the potato slices dried by EH + EPD and FD, and the pores of dried slices by EH + EPD are larger. Ethanol can dissolve components in the cell wall and modify its structure to increase its permeability (Amanor-Atiemoh et al., 2020), while sufficient carbon dioxide entering into potato slices increases puffing power, and EH + EPD-dried samples form more regularly larger pores.

#### Rehydration ratios

**Fig. 3:**
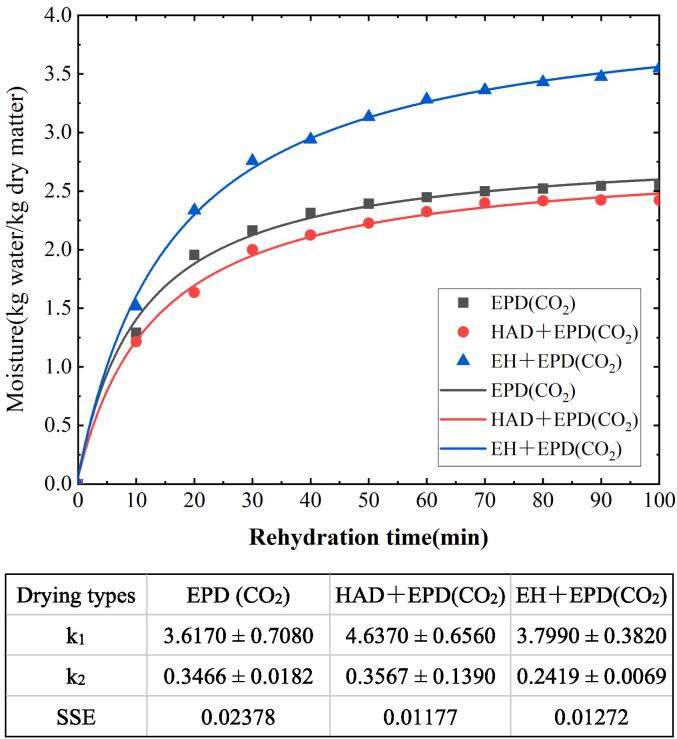
Rehydration kinetics model. Dots are the experimental data. Curves are the Peleg mode. The table is k_1_, k_2_, SSE calculation value.

The rehydration kinetics of potato slices for EPD (CO_2_), HAD + EPD (CO_2_), and EH + EPD (CO_2_) is shown in Fig. 3. Different pretreatment methods will change the cell structures of potatoes during puffing drying, resulting in different rehydration. The lower the *k*_1_ and *k*_2_ values, the higher the water absorption and the equilibrium moisture content by the Peleg model (Eq.8). The HAD + EPD (CO_2_) sample has lower water absorption and water retention capacity (the higher *k*_1_ and *k*_2_ values, respectively), and the EH + EPD (CO_2_) has better water absorption and water retention capacity. Ethanol treatment improves the rehydration process, resulting in better rehydration and water retention (Rojas & Augusto, 2018). The potato starch gelatinization temperature is close to 66 °C (Castanha et al., 2017), and the rehydration process is affected by structural changes during the drying process. Dense or collapsed structures reduce the absorption and water retention capacity (Rojas et al., 2019). The potato has high starch content and structure, and its structure will be changed when it is pretreated with hot air at 60 °C in HAD + EPD (CO_2_), resulting in poor puffing and drying effects, and reducing rehydration. Rojas, M. L, *et al.* observed a decrease in the infrared (IR) rehydration performance after Ultrasound and ethanol pre-treatments (Ethanol + U/S) (Rojas et al., 2019). However, the higher water absorption and water retention capacity of EH + EPD (CO_2_) are due to the fact that the ethanol can change the structure of potato slices to increase its permeability.

### Effects of different conditions on the mass transfer of potato slices

#### Water loss

**Fig. 4:**
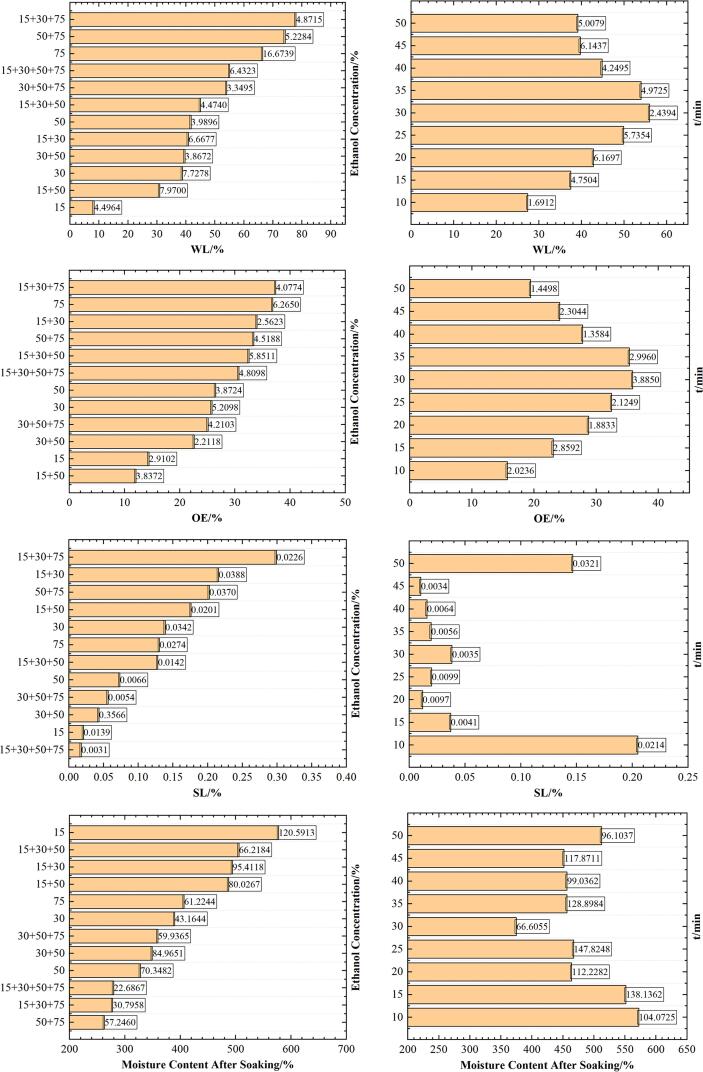
Effects of Ethanol Concentration and time on WL, OE, SL, Moisture Content After Soaking.

The effects of ethanol concentration and soaking time on WL are shown in Fig. 4. The high ethanol concentration needs less time to dry than the low ethanol concentration (da Cunha et al., 2020). Compared with single-stage dehydration, it can be concluded that the higher the concentration of ethanol, the larger the WL; compared with single-stage dehydration, the WL of high-concentration multi-stage dehydration is larger. During the process of ethanol from low to high concentration, the water molecules in the tissue are continuously bound by hydroxyl groups in ethanol, and the moisture in the tissue is slowly removed and replaced by ethanol. As the time when potato slices are immersed in ethanol solution is increased, the low-concentration liquid penetrates into the high-concentration liquid, where ethanol replaces the water inside the potato slices, so the WL is increased. The increase in WL is more pronounced during the initial soak due to the larger osmotic driving force between the fresh sample, and the solution attaches to the sample surface. After a constant period of time, the WL is gradually stabilized. With the adhesion of the penetration, the diffusion rate of water from the inside to the surface of the potato slice becomes smaller, which is diluted by the water that has diffused from the inside, and the WL is decreased gradually and tends to a steady state (Mokhtar et al., 2019).

#### Obtained ethanol

The changes of OE in time and ethanol concentration are shown in Fig. 4. Similar to changes in WL, ethanol is also replaced in the water of the material when the material is dehydrated. The high concentration has more OE than the low ethanol concentration does, and the high concentration step soaks larger than the OE of the single-stage concentration. With the increase of time, OE is first increased and then decreased, because in the dehydration of ethanol, the water inside the material cells will penetrate into the ethanol due to the concentration difference and OE will increase. However, with the increase of time, the internal tissue and the concentration of ethanol solution reach an equilibrium state. This is a dynamic process.

#### Solids loss

The effects of ethanol concentration and soaking time on SL are shown in Fig. 4. There is no significant correlation between SL and ethanol concentration (p greater than 0.05), and SL changes irregularly with the increased soaking time. Da Cunha et al studied that the melons were immersed in 100% ethanol with solid loss, which was larger than 50% SL (da Cunha et al, 2020), confirming that the solid loss of watermelon soaked in high concentration ethanol was greater than that of low concentration ethanol. And it can be seen that 75% is larger than 50% SL from Fig. 4, in line with their findings match. Ethanol can dissolve compounds on the cell wall, which leads to some changes in the cell wall structures, increases permeability, and promotes the loss of larger molecules (solids) (Wang et al., 2018). There are differences in the distribution of starch and other nutrients in different parts of potatoes, so SL will also be different. Potato slices soak in ethanol, and fresh potatoes may have different changes in the internal components during the drying process, and SL will also change irregularly over time. It is worth continuing to study the changing law of SL in the future.

#### Moisture content after soaking

The effects of ethanol concentration and soaking time on the moisture content (dry basis) of potato slices after soaking are shown in Fig. 4. The moisture content after soaking includes WL, OE in potato slices, and the changes of the WL and OE directly affect the change of moisture content. The higher the ethanol concentration, the lower the moisture content, while the higher the concentration of multi-stage dehydration, the lower the moisture content. With the increase of soaking time, the water content is first decreased and then increased.

### Effects of ethanol dehydration and puffing conditions on potato expansion characteristics and quality

#### Effects of WL, OE and SL on expansion characteristics and quality

**Fig. 5:**
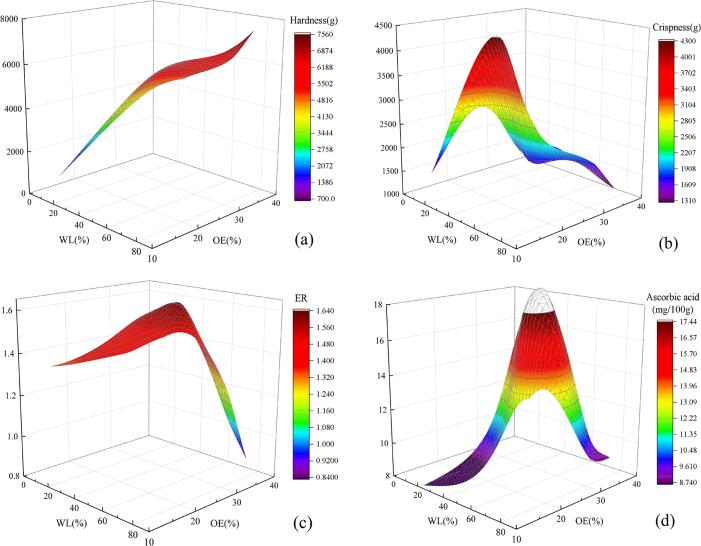
Effect of  WL、OE and SL on expansion characteristics and quality.

The effects of WL and OE on hardness, crispness, expansion ratio and ascorbic acid by soaking potato slices in different ethanol concentrations are shown in Fig. 5. WL and OE have no significant impacts on L* and Δ*E*. SL has no significant effect on hardness, crispness, expansion ratio, ascorbic acid, L*, and Δ*E*. From Fig. 5 (a), (b), (c) and (d), it can be concluded that when WL is increased, the hardness is increased, ascorbic acid, crispness, and expansion ratio are first increased and then decreased; when OE is increased, the hardness is increased and expansion ratio is increased. The hardness, crispness and ascorbic acid are first increased and then decreased. The increase of ethanol concentration & WL will destroy the cell wall. In the process of puffing and drying, the moisture content of the potato slices is decreased, and the hardness and crispness are also increased after drying; when the WL is larger, the hardening phenomenon will occur during the drying process, resulting in the decrease of crispness. During the puffing process, the potato slice needs its internal liquid (water and ethanol) as power. The solubility of carbon dioxide in ethanol is higher compared with water. When the OE inside the potato is increased, more carbon dioxide will dissolve into the potato slices, which will enhance the puffing power and increase the expansion ratio. When the OE is larger, the high puffing power can promote the rapid gasification of water, making it difficult for the cell wall to rupture to form a porous structure, so as to affect the pore structure of the puffed product (Liu et al., 2020), resulting in the reducing of expansion ratio and crispness of potato slices. Carbon dioxide is an acid gas, and the stability of ascorbic acid is improved under acidic conditions. In the puffing and pressurization stage, with the increase of the WL and OE, the moisture inside the potato is decreased, and the ethanol is increased. The dissolution of carbon dioxide in ethanol is more than that in water, and more carbon dioxide dissolves into ethanol inside the potato slices. Ascorbic acid is relatively stable, but when WL and OE are larger, ascorbic acid is easily soluble in ethanol and water, which will cause a small amount of ascorbic acid to dissolve in ethanol and water.

#### Effects of moisture content after soaking on expansion characteristics and quality

**Fig. 6:**
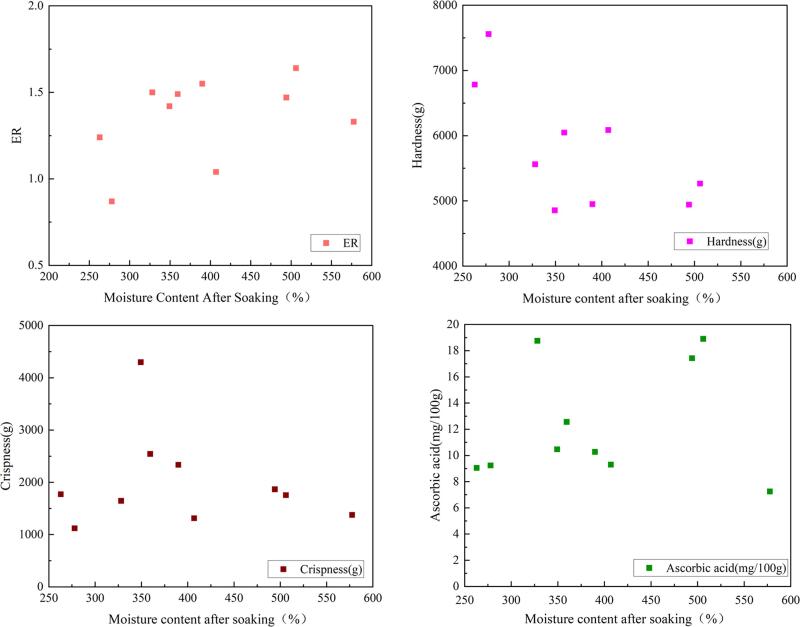
Effect of moisture content after soaking on expansion characteristics and quality.

The moisture content of the potato slices after ethanol soaking affects the expansion ratio, hardness, crispness and ascorbic acid content from Fig. 6. The analysis shows that the moisture content after soaking is increased, the expansion ratio is gradually increased, and the hardness is decreased gradually, the crispness is first increased and then decreased, and there was no significant change in ascorbic acid. The higher the moisture content, the higher the OE, the more CO_2_ dissolved in the potato slices, the stronger the puffing power, and the larger the expansion ratio. During the same drying time, low moisture potato slices have a higher hardness. Potato slices need enough water and ethanol as the puffing medium. As the moisture content of the potato slices is increased after soaking; the puffing power is enhanced, and more porous structures are formed inside, thereby increasing the crispness. When the moisture content reaches a critical value to rise after the value, the moisture content in the potato slices is too high, so that the moisture cannot be completely vaporized, which affects the puffing and reduces the crispness.

#### Effect of puffing pressure and temperature on expansion characteristics and quality

**Fig. 7:**
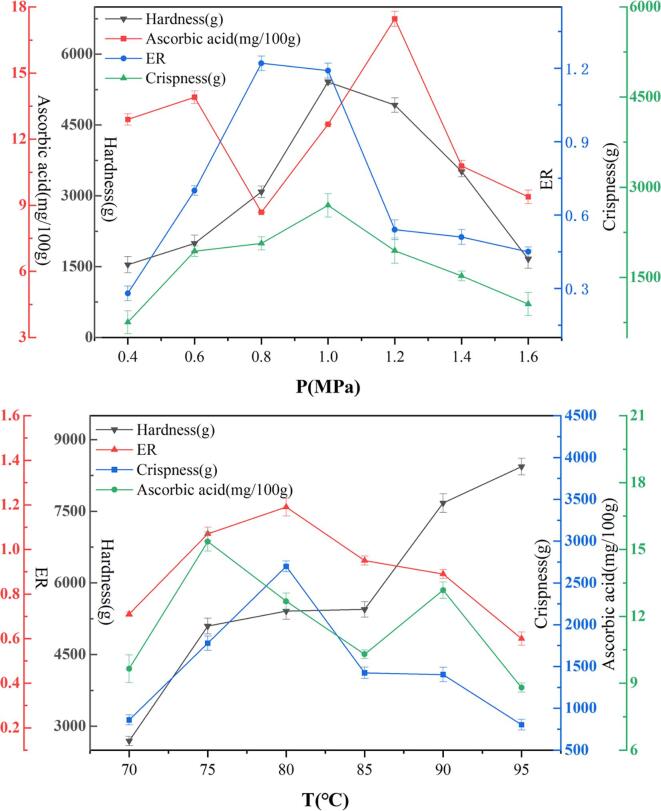
Effect of puffing differential pressure on expansion characteristics and quality.

With the increase of pressure, the expansion ratio, hardness and crispness are first increased and then decreased from Fig. 7. When the pressure difference is low, the puffing power is insufficient during the puffing process, resulting in a low expansion ratio and affecting the sensory quality. When the puffing pressure differences are increased, ethanol can change the structure of the potato slices to increase its permeability, so that the carbon dioxide dissolved in the ethanol is increased, the puffing power is enhanced, and the expansion ratio and crispness become larger. When the pressure difference is too large, there will be too much dissolved carbon dioxide. During the puffing process, the gas carries the expansion medium to destroy its organizational structure and form an internal collapse, resulting in the decrease in the expansion ratio. The studies of Lyu *et al.* showed that the increase exceeded the resistance of the cell porous wall to internal pressure, leading to the destruction of the cell porous wall (Lyu et al., 2021).

With the increase of temperature, the hardness is increased, the expansion ratio and crispness are first increased and then decreased from Fig. 7. As the temperature is increased, the starch inside the potato slices will be gelatinized and become firmer. Crispness is the result of a porous structure formed by air cavities surrounded by crispness structures (La Fuente and Lopes, 2018), and there is a positive correlation between crispness and expansion ratio. After a constant pressure difference is formed in the puffing chamber, the boiling point of ethanol and water is decreased. As the temperature rises, ethanol and water get overheated. During the puffing process, more ethanol and water vaporize instantaneously, which increases the puffing power and the expansion ratio. As it gets higher, the crispness is increased. However, when the puffing temperature is too high, it is not conducive to the dissolution of carbon dioxide in water and ethanol, resulting in the decrease in expansion ratio and crispness.

## Conclusions

(1) The quality and physicochemical characteristics of potato slices by EPD (CO_2_), HAD + EPD (CO_2_), EH + EPD (CO_2_) and FD are compared. FD retains higher ascorbic acid content, color and structure, but lower brittleness and hardness, and poor mouthfeel. In the process of EH + EPD (CO_2_), the solubility of CO_2_ in ethanol is larger than that in water, and more CO_2_ can be dissolved in the puffing medium, so that the puffing power is enhanced, the expansion ratio is increased, and the crispness and hardness are enhanced. EH + EPD (CO_2_) has good structures, colors, and high ascorbic acid contents, and possesses moderate crispness and hardness. Based on the Peleg model, EH + EPD (CO_2_) also has a great ability to absorb water and retain water.
(2) The mass transfer process of potato slices at different ethanol concentration and soaking time is studied. Compared with single-stage dehydration, it can be concluded that the higher the concentration of ethanol, the larger WL and OE. In the meantime, the WL and OE of high-concentration multi-stage dehydration are larger. The drying time of high-concentration ethanol is less than that of low-concentration ethanol. The SL and ethanol concentrations have no significant effects (p greater than 0.05). With the increase of soaking time, SL is first increased and then decreased.
(3) The effects of WL, OE, SL, moisture, puffing pressure difference and puffing temperature on the drying quality and physicochemical properties of potato slices by CO_2_ high pressure and low-temperature puffing drying are studied. When WL is increased, the hardness is increased, while the ascorbic acid, crispness, and the expansion ratio are first increased and then decreased. When the OE is increased, the hardness is increased, while the expansion ratio, crispness, and ascorbic acid are first increased and then decreased. With the increase of moisture content, the expansion ratio is gradually increased, the hardness is gradually decreased, and the crispness is first increased and then decreased. The expansion ratio, hardness and crispness are first increased and then decreased with the increase of pressure. The hardness is increased with the increase of temperature, and the expansion ratio and crispness are first increased and then decreased with the increase of temperature.

## Funding sources

This research did not receive any specific grant from funding agencies in the public, commercial, or not-for-profit sectors.

## CRediT authorship contribution statement

**Yao Niu:** Writing – original draft, Methodology, Formal analysis, Visualization, Software, Investigation, Conceptualization, Data curation, Methodology. **Haifeng Chen:** Methodology, Writing – review & editing, Resources, Supervision, Project administration. **Zifeng Zhang:** Formal analysis, Validation, Data curation. **Yuejin Yuan:** Writing – review & editing, Resources, Supervision. **Shaobo Dong:** Formal analysis, Validation. **Zhuo Xu:** Software.

## Declaration of Competing Interest

The authors declare that they have no known competing financial interests or personal relationships that could have appeared to influence the work reported in this paper.

## Data Availability

Data will be made available on request.
